# Impact of Cardiopulmonary Bypass and Aorta Cross Clamp Time on the Length of Mechanical Ventilation after Cardiac Surgery among Children: A Saudi Arabian Experience

**DOI:** 10.7759/cureus.5333

**Published:** 2019-08-07

**Authors:** Akhter Mehmood, Rashid N Nadeem, M S Kabbani, Altaf H Khan, Omar Hijazi, Sameh R Ismail, Ghassan Shath, Winston W Eng, Shafaq Jawed

**Affiliations:** 1 Pediatrics, Dubai Hospital, Dubai, ARE; 2 Intensive Care Medicine, Dubai Hospital, Dubai, ARE; 3 Cardiac Sciences, King Abdulaziz Medical City, National Guard Hospital Health Affairs, Riyadh, SAU; 4 Bioinformatics, King Abdulaziz Medical City, National Guard Hospital Health Affairs, Riyadh, SAU; 5 Miscellaneous, Performance Center, Omnicell, Inc., Pittsburgh, USA; 6 Surgery, Jinnah Sindh Medical University, Karachi, PAK

**Keywords:** cardiopulmonary bypass time, aorta cross clamp time, prolong mechanical ventilation, hospital length of stay

## Abstract

Aim

Several factors determine the perioperative outcome besides the nature of the congenital heart defect. Prolonged mechanical ventilation (PMV) is a major factor that determines mortality, length of stay (LOS), residual disability, and other functional outcomes. We aim to determine the clinical variables predicting PMV and LOS in hospital, and specifically the impact from the duration of cardiopulmonary bypass (CPB) and aortic cross-clamp (ACC).

Method

We conducted a retrospective review of the medical records of 413 children consecutively admitted to the Pediatric Cardiac Intensive Care Unit (PCICU) in one year at a single center. We collected demographic information (e.g., age, gender, and weight), perioperative variables, clinical outcomes, length of mechanical ventilation, high-frequency ventilator use, and mortality. We used logistic regression to analyze the data. PMV was defined as mechanical ventilation for longer than seven days.

Results

A total of 410 records were included in our study. We found no statistically significant association between CPB time and mechanical ventilation days. Forty-seven children had PMV, 362 did not have PMV. We found no statistically significant association between CPB time and mechanical ventilation days after adjusting for covariates. Reanalyzing the data with PMV defined as longer than four days produced the same results. Using a regression model to assess the variables via the least absolute shrinkage and selection operator for feature selection, we found no statistically signiﬁcant association between ACC time and mechanical ventilation days after adjusting for covariates.

Conclusion

According to our results, CPB and ACC time are not associated with PMV or prolonged hospital LOS.

## Introduction

The prevalence of congenital heart disease in children is 3.7/1,000 live births in the USA [1]. A British study reported that among 160,480 children born alive between 1960 and 1969 in Liverpool, 884 patients had structural congenital heart disease (i.e., 5.5/1000 live births) [2]. From 1979 through 1997, mortality from heart defects (in patients of all ages) declined from 2.5 to 1.5 per 100,000 population [3]. Recent advances in surgical technique and medical care in the last five years have improved the perioperative survival of pediatric patients with congenital heart disease to about 96% [4]. Perioperative risk categorization of children, depending upon the type of congenital heart disease and performed surgery (categories one to six, with increasing complexity) is predictive of increasing mortality [5, 6]. There are several factors determining perioperative outcomes besides the nature of the congenital heart defect and risk category. Age, gender, number of surgeries on the same patient, the volume of the surgeries performed by hospitals, duration of cardiopulmonary bypass (CPB), aortic cross-clamp (ACC) time, postoperative organ dysfunction, and renal failure may play a significant role [7-9]. Mechanical ventilation (MV) for longer than seven days (i.e., prolonged MV [PMV]) is a major factor in determining mortality, length of stay (LOS), residual disability, and other functional outcomes. The literature on these factors in determining PMV and prolonged stay in the intensive care unit (ICU) or hospital is generally minimal and largely unknown for the patients in the Kingdom of Saudi Arabia (KSA). We aim to determine clinical predictors of outcomes in this pediatric population in general and determinants of PMV and LOS in hospital, specifically, the impact of the duration of CPB and ACC, at a medical center in the KSA.

## Materials and methods

We conducted a retrospective review of the medical records of all consecutively admitted children to the Pediatric Cardiac Intensive Care Unit (PCICU) for one year. We excluded incomplete records from the study (n=3). A total of 410 patient records were included in our review. Patients had undergone cardiac surgeries and stayed in the PCICU of the Cardiac Sciences Department at King Abdul-Aziz Cardiac Center.

We reviewed and collected demographic data (e.g., age, gender, and weight) and preoperative variables (e.g., diagnosis, name of the clinical syndrome, cardiac functions, risk category and history of pulmonary hypertension). We also collected comorbidities such as gastroesophageal reflux disease (GERD) and airway anomalies. We reviewed intraoperative variables, including CPB time, ACC time, and use of inhaled nitrous oxide. Postoperative variables collected included infection, pneumonia, catheter-related infections, catheter-associated urinary tract infections (UTI), noninfectious pulmonary complications, pneumothorax, chylothorax, cardiomyopathy, shock, mediastinitis, failure of sternal wound closure, surgical site infection, necrotizing enterocolitis, bacteremia, blood transfusion, acute renal failure, use of total parenteral nutrition (TPN), PCICU and hospital LOS, extubation failure, interventions (laser procedures for subglottic bands), length of MV, use of high-frequency oscillatory ventilator (HFOV), and mortality.

We utilized RACHS-1 (risk adjustment for congenital heart disease) based on a surgical risk complexity scoring system that categorizes surgical procedures according to the severity of the cardiac lesion and the complexity of the cardiac repair. There were six categories, with one representing the lowest surgical risk and six representing the highest surgical risk [6].

Statistical analysis

Total MV days was initially treated as count data. After assessing for over-dispersion, a negative binomial regression model with the least absolute shrinkage and selection operator (LASSO) was fitted. First, potential covariates were identified by retaining those with λ coeﬃcient within one standard error of the minimum, using all 28 possible clinical confounding variables collected. While adjusting for these covariates, the relationship between MV days and CPB time was assessed.

Additionally, MV was subcategorized into PMV (MV for longer than seven days) and non-PMV (MV for seven or fewer days). We also compared MV for over four days with MV for four or fewer days in separate analyses. After creating this binary variable, logistic regression with LASSO was fitted to determine the significant covariates and subsequently assess the redefined MV variable with CPB time for both PMV definitions, respectively. All analyses were performed using R [10].

## Results

We reviewed the records of 413 children admitted to the PCICU for cardiac surgery. After excluding three patients’ records for incomplete data, our ﬁnal sample size was 410 patient medical records. Our sample composition is presented in Table 1.

**Table 1:** Continuous variables PCICU - Pediatric Cardiac Intensive Care Unit

Variable	Mean	Standard Deviation
Age (months)	21.75	28.7
Weight (kg)	8.90	6.47
Bypass time (minutes)	94.46	53.25
Cross-clamp time (minutes)	64.32	38.55
PCICU length of stay (days)	8.43	14.71
Hospital stay (days)	23.79	28.28
Mechanical ventilation (days)	3.84	7.66

Effect of cardiopulmonary bypass time on prolonged mechanical ventilation

**Figure 1:**
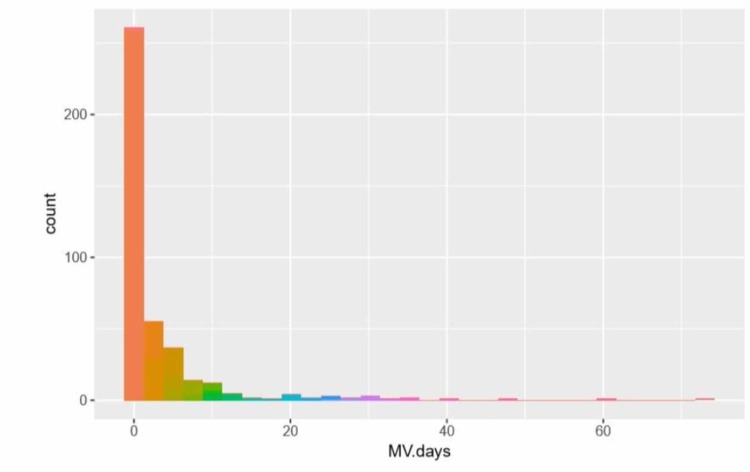
Distribution of mechanical ventilation days MV - mechanical ventilation

Determinants of PMV days as a function of other variables: After running the negative binomial regression with LASSO with 28 possible variables, we found PCICU LOS to have the λ coeﬃcient within one standard error of the minimum (Figure 1).

**Figure 2:**
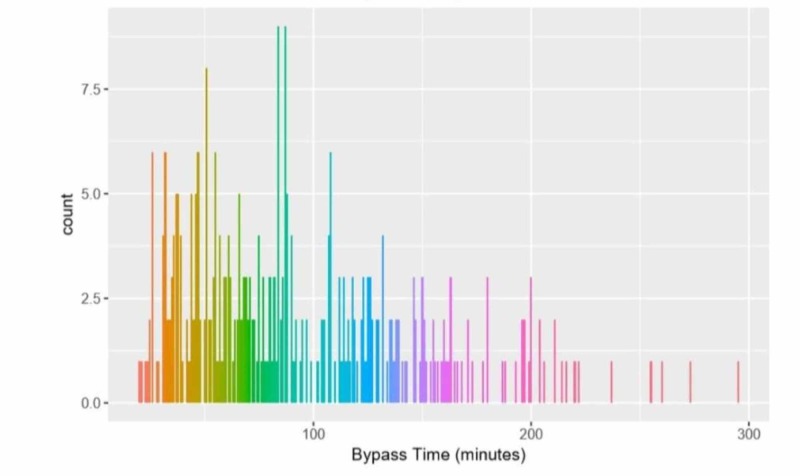
Distribution of cardiopulmonary bypass time

As a result, we decided to retain these variables as a covariate and include them in the subsequent analyses. For the logistic regression model to assess the relationship of CPB time on outcome variable PMV, we use the covariates gained via LASSO for feature selection. We found no statistically significant association between CPB time and MV days (Figure 2) after adjusting for covariates (p=0.11).

MV lasting longer than seven days was considered PMV, and we sorted the data into two categories: PMV (n=47) and non-PMV (n=362). We reran the model to see if CPB time and ACC time predict PMV. We found significant λ for PCICU stay (λ=0.09), TPN (λ=1.16), HFOV (λ=1.72), interventions (λ=-0.49), heart dysfunction (λ=0.42), bloodstream infection (BSI; λ=0.129), pneumonia (λ=2.17), and UTI (λ=1.066). These variables were included in the final model of CPB time logistic regression model (split on MV for longer than seven days) which showed no statistically significant association between CPB time and MV days after adjusting for covariates (p=0.17).

We repeated our analysis using an altered definition of PMV as longer than four days (reduced from seven days). Seventy-three patients had PMV, and 334 did not have PMV. Factors with significant λ were included in CPB time logistic regression model (split on MV > 4 days) which showed no statistically significant association between CPB time and MV days after adjusting for covariates (p=0.65).

Effect of aorta cross-clamp time on prolonged mechanical ventilation

**Figure 3:**
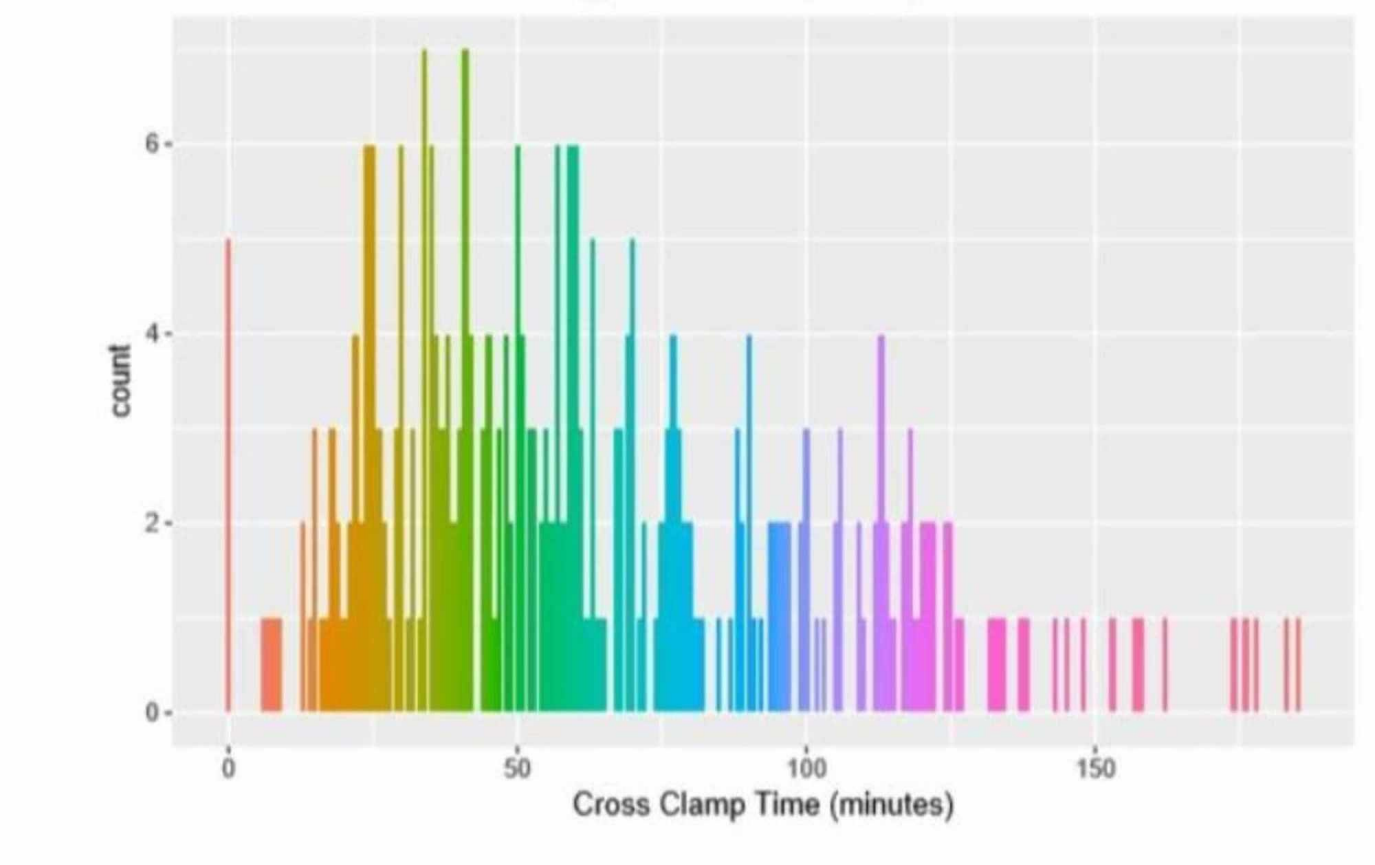
Distribution of aortic cross-clamp time

Because there is a similar relationship between CPB time and ACC time, we ran another model assessing the relationship between PMV days and ACC time after adjusting for covariates (Figure 3).

For this model, we treated MV days as the outcome variable and assessed its relationship with ACC time. Using a logistic regression model with the variables gained via LASSO for feature selection, we found no statistically signiﬁcant association between ACC time and MV days after adjusting for covariates (p=0.40).

Effect of cardiopulmonary bypass time on the length of stay in hospital

**Figure 4:**
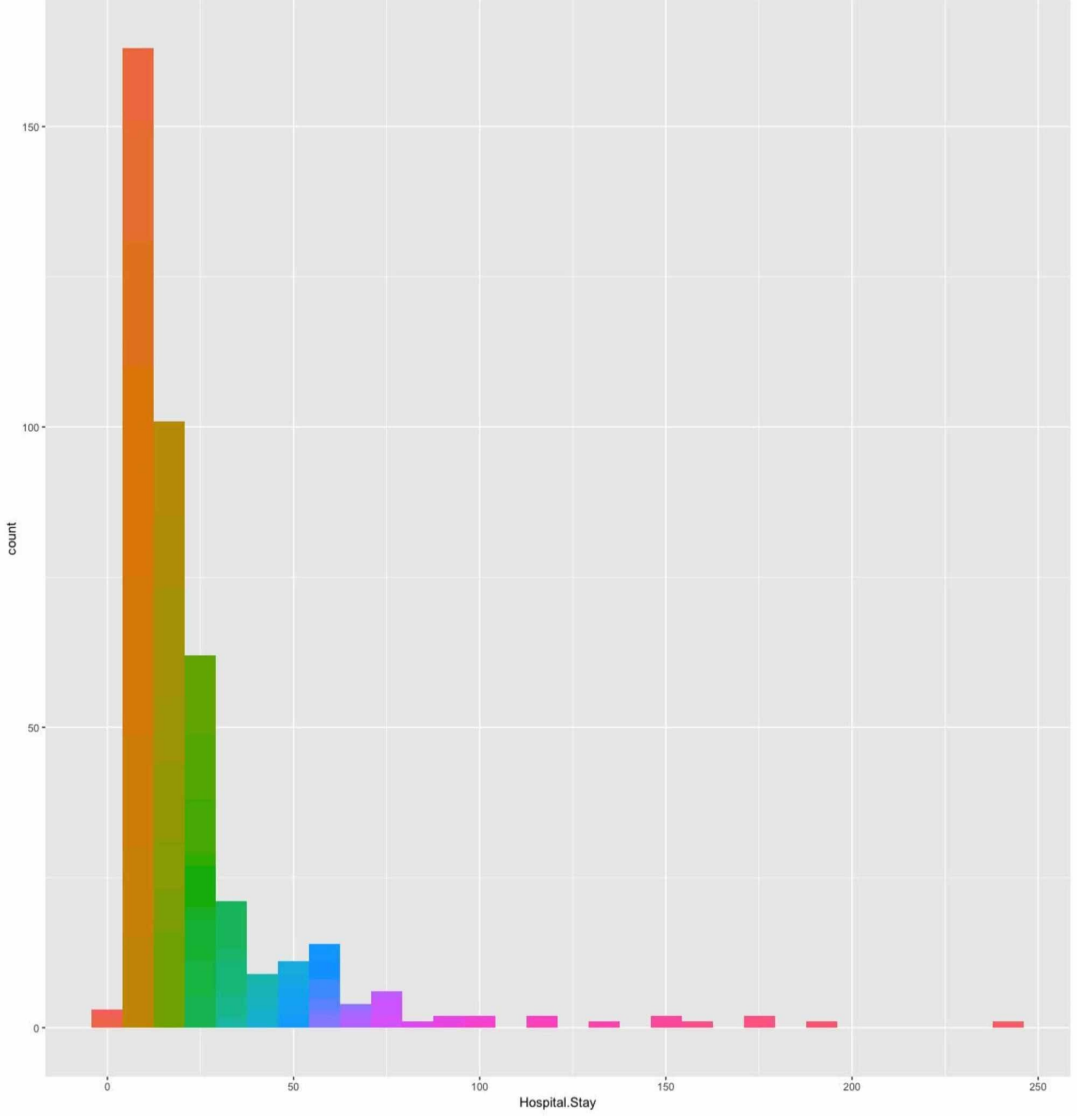
Distribution of length of stay

We assessed whether the CPB time predicts LOS. In this model, we found significant λ for gender (λ=0.027), risk category (λ=0.193), PCICU LOS (λ=0.172), MV days (λ=0.0012), pulmonary hypertension (λ=0.221), extubation failure (λ=0.153), airway anomaly (λ=0.118), pulmonary complications (λ=0.269), GERD (λ=0.053), surgical site infection (SSI; λ=0.305), BSI (λ=0.571), and pneumonia (λ=0.050). These variables were included in the final model of CPB time negative binomial regression, which showed no statistically significant association between CPB time and LOS after adjusting for covariates (p=0.36; Figure 4).

Effect of aorta cross-clamp time on the length of stay in hospital

We found significant λ for gender (λ=0.027), risk category (λ=0.193), PCICU LOS (λ=0.172), MV days (λ=0.0012), pulmonary hypertension (λ=0.221), extubation failure (λ=0.153), airway anomaly (λ=0.118), pulmonary complications (λ=0.269), GERD (λ=0.053), SSI (λ=0.305) ), BSI (λ=0.571), and pneumonia (λ=0.050). These variables were included in the final model of ACC time negative binomial regression, which showed no statistically significant association between ACC time and LOS after adjusting for covariates (p=0.89).

## Discussion

CPB time is not a significant determinant of the duration of postoperative MV in children with cardiovascular surgeries. Polito et al. also found no association of CPB time to PMV [11]. They used an arbitrary definition of PMV as MV lasting longer than seven days. We used the same criterion for MV and found no significant association between PMV (>7 days) and CPB time. Another study by Szekely et al. found an association between PMV and CPB time [12]. Their definition of PMV used a shorter threshold than our study, defining PMV as MV for longer than four days (rather than seven in our design). We performed a second analysis using Szekely’s definitions of PMV and found no association between PMV, CPB time, and ACC time. The difference in results is likely due to a difference in methodologies between the studies and the incorporation of different confounding variables in their model compared to ours; we used CPB time as a continuous variable while they categorized CPB as a risk factor if lasting longer than 30 minutes. We also included UTI, pneumonia, and BSI as confounding variables which may have an impact on PMV and LOS.

We found that CPB and ACC do not predict LOS. Other studies report that CPB time is a risk factor influencing ICU LOS in both children and adults [13-15]. The difference in results may be from different methodologies and different sets of confounding variables. For example, infection, UTI, pneumonia, BSIs, and glycemic control are well-known risk factors that determine LOS in the ICU and hospital and affect mortality, survival and other outcomes in the medical and surgical patient population [16-19]. If a study does not incorporate these factors in the adjustment model, it may attribute a larger association to CPB or ACC times to outcomes of LOS and PMV.

If the confounding variables are categorized differently, they mitigate the contribution of those factors as a determining factor. To prove this concept, we reran our model with surgical risk categories one to six as six different categories (instead of two categories, <3 and >3) which results in a positive association of CPB and ACC with PMV and LOS, although it may be the result of a failure to incorporate the contribution of higher surgical risk categories as a determinant of morbidity and LOS. Similarly, categorizing CPB time as 30-minute categories as a risk factor instead of continuous variables may have an amplifying effect on its contribution to LOS and PMV.

With recent advances in technology regarding the improvement in gas exchange membrane quality and machine interface surfaces, the risk documented in older studies may have improved, as the mechanism of insult imposed by CPB or ACC may be from the activation of complement leukocytes and endothelial cells secreting cytokines, proteases, arachidonic acid metabolites, and oxygen-free radicals. Leukocyte adhesion to the microvascular endothelium, leukocyte extravasation, and tissue damage can cause intrapulmonary shunt and a decrease in pulmonary compliance, which leads to acute lung injury and acute respiratory distress syndrome, requiring the support of MV [20].

By applying a different methodology and multiple ways of categorization, we documented a greater impact from confounding variables (which were previously documented to impact outcomes) upon outcome measures which could have been incorrectly attributed to CPB time or ACC time.

Our study had several limitations. The retrospective design of this study provides a lesser quality of evidence in general and cannot exclude any causal relationships. However, there are no large prospective trials available on this issue. We were not able to assess mortality or survival, as only five patients died, which is a small number for any calculation. This reflects recent improvements in surgical and medical care - mortality may not be a valid outcome measure for some disorders. This also highlights the dynamic nature of CPB and ACC as risk as technology continues to improve. Lastly, our small sample size did not allow us to measure each variable’s impact on outcomes or isolate each variable’s effect on CPB and ACC time as well as with each other.

## Conclusions

Cardiopulmonary bypass time and aorta cross-clamp time are not associated with prolonged mechanical ventilation and length of stay in hospital. It appears that clinical factors other than CPB time and ACC time may play a bigger role in determining clinical outcomes. Therefore better patient selection, surgical technique, and perioperative care may improve clinical outcomes. Larger prospective studies to address this issue are urgently warranted. 

## References

[1] Ferencz C, Rubin JD, Mccarter RJ (1985). Congenital heart disease: prevalence at livebirth: the Baltimore-Washington Infant Study. Am J Epidemiol.

[2] Dickinson DF, Arnold R, Wilkinson JL (1981). Congenital heart disease among 160 480 liveborn children in Liverpool 1960 to 1969. Implications for surgical treatment. Heart.

[3] Boneva RS, Botto LD, Moore CA, Yang Q, Correa A, Erickson JD (2001). Mortality associated with congenital heart defects in the United States: trends and racial disparities, 1979-1997. Circulation.

[4] Gross RE (1939). Surgical management of the patent ductus arteriosus: with summary of four surgically treated cases. Ann Surg.

[5] Moller JH, Dwan P (1998). Surgery of congenital heart disease: Pediatric Cardiac Care Consortium, 1984-1995.

[6] Larsen SH, Pedersen J, Jacobsen J, Johnsen SP, Hansen OK, Hjortdal V (2005). The RACHS-1 risk categories reflect mortality and length of stay in a Danish population of children operated for congenital heart disease. Eur J Cardiothorac Surg.

[7] Oster ME, Strickland MJ, Mahle WT (2011). Racial and ethnic disparities in post-operative mortality following congenital heart surgery. J Pediatr.

[8] Hannan EL, Racz M, Kavey R-E, Quaegebeur JM, Williams R (1998). Pediatric cardiac surgery: the effect of hospital and surgeon volume on in-hospital mortality. Pediatrics.

[9] Li S, Krawczesk CD, Zappitelli M (2011). Incidence, risk factors, and outcomes of acute kidney injury after pediatric cardiac surgery-a prospective multicenter study. Crit Care Med.

[10] R Core Team (2013). R: A language and environment for statistical computing.

[11] Polito A, Patorno E, Costello JM (2011). Perioperative factors associated with prolonged mechanical ventilation after complex congenital heart surgery. Pediatric Critical Care Medicine.

[12] Szekely A, Sapi E, Kiraly L, Szatmari A, Dinya E (2006). Intraoperative and postoperative risk factors for prolonged mechanical ventilation after pediatric cardiac surgery. Paediatr Anaesth.

[13] Izabela PK, Magdalena PP, Wojciech K, Moll JJ (2011). Predictors of long intensive care unit stay following cardiac surgery in children. Eur J Cardiothorac Surg.

[14] Brown KL, Ridout DA, Goldman AP, Hoskote A, Penny DJ (2003). Risk factors for long intensive care unit stay after cardiopulmonary bypass in children. Crit Care Med.

[15] Gillespie M, Kuijpers M, Van Rossem M, Ravishankar C, Gaynor JW, Spray T, Clark B (2006). Determinants of intensive care unit length of stay for infants undergoing cardiac surgery. Congenit Heart Dis.

[16] Arozullah MA, Khuri SF, Henderson WG, Daley J (2001). Development and validation of a multifactorial risk index for predicting postoperative pneumonia after major noncardiac surgery. Ann Intern Med.

[17] Hurley JC, Guidet B, Offenstadt G, Maury E (2012). Endotoxemia and mortality prediction in ICU and other settings: underlying risk and co-detection of gram negative bacteremia are confounders. Critical Care.

[18] Mitchell BG, Fergusonc JK, Andersona M, Seara J, Barnettf A (2016). Length of stay and mortality associated with healthcare-associated urinary tract infections: a multi-state model. J Hosp Infect.

[19] Marchant M, Viens N, Cook C, Vail T, Bolognesi M (2009). The impact of glycemic control and diabetes mellitus on perioperative outcomes after total joint arthroplasty. JBJS.

[20] Asimakopoulos G, Smith PLC, Ratnatunga CP, Taylor KM (1999). Lung injury and acute respiratory distress syndrome after cardiopulmonary bypass. Ann Thorac Surg.

